# Parents' Perspectives on Access to Pediatric Rare Disease Cross-Border Clinical Trials in Europe: Experiences of Language Inclusion and Preferences

**DOI:** 10.1007/s43441-026-00942-y

**Published:** 2026-03-18

**Authors:** Begonya Nafria, Segolene Gaillard, Martine Dehlinger-Kremer, Pamela Dicks, Ricardo M. Fernandes, Lucy Bray, Barbara E. Bierer, Bob Phillips

**Affiliations:** 1 Institut de Recerca Sant Joan de Déu, Head of the Patient Engagement in Research Department, Santa Rosa 39-57, 08950 Esplugues de Llobregat, Spain; 2 Universitat Internacional de Catalunya2, PhD Student, Immaculada 22, 08017 Barcelona, Spain; 3 European Young Person’s Advisory Groups Network – eYPAGnet, Barcelona, Spain; 4 CIC 1407, Inserm, Kids France, CHU-Lyon, Lyon, France; 5 Laboratoire de biométrie et biologie évolutive, Université Lyon France, Lyon, France; 6 European Young Person’s Advisory Groups Network – eYPAGnet, Lyon, France; 7 UCB Biosciences, Monheim, Germany; 8 NHS-NRS Children, NHS Grampian, Royal Aberdeen Children’s Hospital, Aberdeen, UK; 9 European Young Person’s Advisory Groups Network – eYPAGnet, Aberdeen, UK; 10 Clinical Pharmacology and Therapeutics, Faculdade de Medicina, Universidade de Lisboa, Lisbon, Portugal; 11 STAND4Kids, Associação para a Investigação e Desenvolvimento da Faculdade de Medicina, Lisbon, Portugal; 12 Faculty of Health, Social Care and Medicine, Edge Hill University, Ormskirk, UK; 13 Multi-Regional Clinical Trials Center of Brigham and Women’s Hospital and Harvard, Boston, Massachusetts USA; 14 Division of Global Health Equity, Department of Medicine, Brigham and Women’s Hospital, Boston, MA USA; 15 Department of Medicine, Harvard Medical School, Boston, Massachusetts USA; 16 Centre for Reviews and Dissemination, University of York, York, UK

**Keywords:** Cross-border clinical trials, Translation, Mother tongue, Preferred language, Informed consent process, Patient reported outcomes measures (PROMs), Quality of life scales, Parents’ preferences

## Abstract

**Introduction:**

Access to cross-border clinical trials may represent the sole therapeutic option for children living with rare diseases for which no approved medicines exist. Many children are excluded from participation in trials due to language restrictions. There are insufficient comprehensive analyses of the experiences and preferences of parents across Europe concerning participation and exclusion of their child in international clinical trials, particularly regarding language support during enrollment in cross-border clinical research studies.

**Methods:**

An anonymous online survey was designed and translated into 22 official European languages to collect data from parents of children living with a disease across Europe. The survey included five sections: (1) sociodemographic information; (2) experience participating in a clinical trial; (3) experience in cases where the patient was unable to take part in a study abroad; (4) experience participating in a clinical trial abroad; and (5) preferences regarding decentralized trial options.

**Results:**

1,436 responses were analyzed from parents across 34 European countries. Key findings: 55.7% of the parents reported being able to communicate in English. 10.7% had prior clinical trial experience, of whom 30.1% traveled abroad to enable their child to participate. Among those reporting being excluded from cross-border trials, 34.7% cited language barriers or country of residence as the reason. Most families expressed a strong willingness to accept decentralized trial options, regardless of where the study may be conducted.

**Conclusions:**

Accommodating language translation to permit participation in a clinical trial abroad is feasible. While a significant percentage of caregivers of pediatric patients in Europe could communicate in English, approximately one-third of those excluded from clinical trials cited language barriers or country of residence as the reason. When translation was required, the most commonly offered solution was the use of professional interpreters, an accommodation that could enable broader patient participation in essential research.

**Supplementary Information:**

The online version contains supplementary material available at 10.1007/s43441-026-00942-y.

## Introduction

Neither the Clinical Trials Regulation (EU No 536/2014) [1] nor the Paediatric Regulation (EC No 1901/2006) [2] regulates cross-border access to pediatric clinical trials in Europe; the number of European Member States involved per trial is limited, averaging 3.1 member states per trial for commercial sponsors and 1.2 for non-commercial sponsors between 2005 and 2020 [3]. Given that most diseases affecting pediatric patients are rare or ultra-rare, it is, therefore, unlikely that a patient will have access to a clinical trial locally. Rare conditions in Europe are those with a prevalence threshold of fewer than 1 in 2,000 people [4]. This unmet need underscores the imperative to facilitate cross-border access in Europe, given that clinical trials often offer access to novel therapeutic approaches when no effective treatment exists.

The inclusion of international patients travelling from abroad in a clinical trial is at the discretion of the study sponsor; supporting their inclusion, however, can be challenging, involving not only travel, accommodation, and other needs but also language accessibility. In some studies, this may simply involve translating patient-facing documents, as quality-of-life scales and other patient/caregiver-reported outcome tools may already be validated in the patient’s native language. In other cases, the absence of validated data collection tools for patients and caregivers in specific languages can be a limiting factor. Despite these challenges, there are reported cases [5, 6] where, in the absence of validated tools in the patient’s or caregiver’s native language, professional translation was arranged to facilitate the enrolment of international patients.

Participation in a pediatric clinical trial affects the entire family, as at least one caregiver is typically required to accompany their child throughout the study. When the clinical trial takes place abroad, the patient’s siblings and/or the second caregiver can be impacted. In many cases, even when the trial is conducted in the patient’s country of residence, one caregiver often has to reduce or to give up his/her professional activity [7, 8] depending on the intensity and duration of study-related procedures.

Given the significant burden that participation in a pediatric clinical trial places on families, we conducted an anonymous, Europe-wide survey targeting caregivers of children living with a rare disease. This survey was designed to assess the lived experience of families participating in clinical trials, whether conducted in their country of residence or abroad. Importantly, the survey included questions for all respondents, regardless of prior trial participation, to explore their potential willingness to enroll in a clinical trial abroad.

The primary objective of this study was to examine the experiences and preferences of parents based in Europe regarding access to cross-border clinical trials for their child with a rare disease. The findings of this research are intended to inform recommendations to facilitate the language accommodation and improve cross-border access to pediatric clinical trials in Europe. This research is has ben conducted by a Working Group within the European Network of Paediatric Research at the European Medicines Agency (Enpr-EMA) [9]. This working group aims to assess the current landscape of multi-regional and cross-border pediatric clinical trials in Europe, with particular attention to the impact of language-related discrimination.

## Materials and Methods

### Design

A 51-question online, anonymous survey was designed to collect data from caregivers of children living with a rare disease across Europe. It was not required to have prior experience in clinical trial participation to be eligible for inclusion in the survey. For those caregivers whose child had participated in a trial, caregivers were asked to describe how language accommodation was implemented. The survey also examined the prospective acceptability of digital and decentralized elements in trial design.

The survey was structured into several sections with closed-ended questions, some of which were completed based on the respondent’s responses: (1) sociodemographic information (e.g., profile of the respondent, and age, patient’s age, country of residence, native language, language proficiency, etc. but without collection of personal or identifiable data); (2) experience in participating in a pediatric clinical trial; (3) experience in situations where the patient was unable to participate in a clinical trial abroad; (4) experience participating in a clinical trial abroad (e.g., consent and assent procedures, health data reporting questionnaires, etc.); and (5) preferences regarding cross-border access to clinical trials and the inclusion of decentralized and digitalized trial options based in a Likert scale. Caregivers responded to this section by considering two scenarios: (1) a study conducted in their country of residence and (2) access to a cross-border trial. This allowed us to compare whether decentralization (e.g., home nursing) and digitalization (e.g. telemedicine), are more favorably received when trials require international travel. (See Supplementary Information- Appendix 1).

The online survey was designed using Qualtrics software, which allows the implementation of the same questionnaire in multiple languages, all accessible through a single link. The tool was designed to allow reporting only once per device, based on the unique site visitor function that identifies IP addresses and cookies, preventing users from attempting to complete the survey multiple times.

### Survey Review Process

Three different advisory boards reviewed the structure and questions of the English version of the survey designed for the study. The boards consisted of (1) six professionals in the field of clinical research and patient involvement, (2) seven parents of children living with a rare disease, and (3) five patient experts/professionals from patient organizations from different European countries. A total of 18 people reviewed the master version of the survey in English.

### Languages

The survey was translated into 22 official European languages by 44 volunteers: 22 who performed the translations and 22 who reviewed them. The survey was made available in the following languages: Bulgarian, Croatian, Czech, Danish, Dutch, Estonian, English, Finnish, French, German, Greek, Hungarian, Italian, Maltese, Norwegian, Polish, Portuguese, Romanian, Serbian, Slovenian, Spanish, and Slovenian. All volunteer translators were either patient experts or professionals in the medical field, with a high level of proficiency in English. The selection of translators was based on their background to ensure that each question was properly understood in English and accurately translated into the volunteer’s native language.

### Dissemination

The survey was disseminated through multiple channels, including direct email messages to patient organizations (sent in several rounds), publishing on professional social media platforms and through patient organizations profiles (LinkedIn, Twitter [now X], and Instagram), messages in patient organizations WhatsApp groups, and newsletter entries (e.g., Orphanet, CIBERER, FEDER, among others). Enpr-EMA also disseminated the survey across all the patient organizations part of the database managed by their Patient Engagement Department at the European Medicines Agency.

Three webinars were organized to introduce this research to patient organizations: (1) 11th of September 2023, conducted in Spanish, (2), 11th of September 2023, conducted in English and addressing the broad community of patient organizations working in Europe, and (3) 25th of September 2023, conducted in French, supported by one member of the Enpr-EMA working group, and addressing patient organizations established in France.

The dissemination period ran from July 16, 2023, to April 7, 2025.

### Ethics Committee Approval

This research project is part of a European initiative, conducted by a Working Group at the European Network of Paediatric Research at the European Medicines Agency (Enpr-EMA). The project and survey were approved by the Ethics Committee of the Institut de Recerca Sant Joan de Déu on March 9, 2023 (code PIC-40-23).

### Analysis

The data was downloaded as a single.xls file. After data curation, a total of 1,436 responses were analyzed using Excel’s statistical descriptive functions (mean, frequency, average, standard deviation, etc.). The same software was used to generate tables and figures.

## Results

### Demographics of Respondents

The survey received 2804 response attempts, of which 1,436 were eligible for analysis as they were fully completed. Incomplete survey responses were defined as those in which respondents abandoned the online survey before reaching the final question. The survey system recorded all responses as well as the percentage of questions completed.

Of the respondents, 80.4% were mothers (n = 1,155), 15.8% were fathers (n = 226), 2.4% were other types of caregivers (n = 35), and 1.4% were young adult patients, all of whom were ≥ 18 years old (n = 20) (Table 1). As the majority of survey respondents were parents, for the purposes of this analysis we will henceforth refer parents as the primary caregivers who contributed most substantially to this study, although the data reported by other types of caregivers and young adult patients (3.8%) is included in the analysis.

**Table 1:** Profile of the survey respondents

Who replied to the survey?	n =	%
Mother	1155	80.4
Father	226	15.8
Another caregiver	35	2.4
Young adult patient	20	1.4
Totals	1436	100

The mean age of respondents, excluding young adult patients, was 42.7 years (SD = 7.4). The mean age of the patients represented in the survey responses was 8.7 years, with an age range from 2 months to 18 years.

88.6% of respondents lived in the country where the patient was born (n = 1,272); 3.6% (n = 52) of those living abroad held permanent residency and dual nationality; and 3.3% (n = 47) were permanent residents with immigrant status (See Supplementary Information).

Regarding the patients’ medical conditions, 96.5% (n = 1,386) of respondents reported that their child had a rare disease, while 3.5% (n = 50) indicated they didn’t know [10]. All respondents provided information about the patient’s diagnosis. The most frequently reported disease was phenylketonuria, with 24 responses, representing only 1.6% of the sample. In contrast, 1,295 different rare diseases were each reported by only one family (90.2%). Among those who indicated uncertainty, a review of the data revealed that only one parent reported a diagnosis of autism, which is not considered a rare disease in children and removed from the dataset. Based on these findings, we can confirm that the dataset analyzed in this study represents parents of children living with a rare disease.

Parents were also asked to report their country of birth, their child’s country of birth, and their current country of residence. In all three cases, Spain and France were the most frequently represented, together accounting for approximately half of the responses. This situation reflects the influence of the survey’s dissemination by the principal and corresponding author in Spain, as well as by one of the co-authors in France. Tables 2, 3, and 4 present data from the five most represented countries for these questions. The complete dataset is available as Supplementary Information.

**Table 2:** Country where the respondent was born

Country	N =	%
Spain	594	41.4
France	114	7.9
Other	112	7.8
Slovenia	85	5.9
Poland	84	5.8
Subtotal	989	68.8

**Table 3:** Country where the patient was born

Country	N =	%
Spain	609	42.4
France	109	7.6
Slovenia	88	6.1
Other	81	5.6
Poland	71	4.9
Subtotal	958	66.6

**Table 4:** Country of residence

Country	N =	%
Spain	619	43.1
France	112	7.8
Slovenia	92	6.4
Portugal	79	5.5
Poland	78	5.4
Subtotal	980	68.2

### Mother Tongue and Competency in Other Languages

The online survey was completed in 21 different European languages, and the three most frequent were Spanish (44.7%, n = 642), English (10.0%, n = 144), and French (8.6%, n = 123). The Serbian version, while offered as a translation, did not receive any responses. The most common native (first) languages of the respondents were Spanish (37.9%, n = 545), French (8.8%, n = 124), and Catalan (6.3%, n = 90)^1^^♣^ (see Supplementary Information).

Several questions about language competence in languages other than the respondents’ native language were included. Of the 1436 respondents, 800 (55.7%) reported being able to communicate in English, with a self-reported proficiency level of intermediate (38.7%, n = 310), advanced (36.7%, n = 294), native (13.9%, n = 111), and beginner (10.6%, n = 85).

Respondents were asked to assess the patient’s ability to speak English. Only 14.7% (n = 211) were able to speak the language fluently. 85.0% (n = 1,221) of the patients could not speak English, and four respondents (0.3%) did not respond to this question. Among those who were reported being able to speak English, 30.8% (n = 65) were native speakers, 29.4% (n = 62) were intermediate, 24.3% (n = 51) advanced, and 15.6% (n = 33) beginner.

38.1% of parents (n = 547) reported the ability to understand and make themselves understood in a language other than English and their native language. The three most spoken languages reported were: Spanish (30.3%, n = 166) French (28.7%, n = 157) and Italian (11.9%, n = 65). Regarding the patients, as reported by their parents, only 16.6% were able to speak a language other than their native language or English.

### Experience as a Participant in a Pediatric Clinical Trial

Thirteen questions were presented to the parents of pediatric patients to analyze the experience of their child participating in clinical trials. 10.6% of the families had previous experience participating in a clinical trial (n = 153), while 89.3% had never participated in a clinical trial (n = 1,283). At the time the families reported this data, 46 (30.1%) were actively participating in a clinical trial.

Among the 153 families with prior experience participating in a clinical trial, 67.9% (n = 104) had participated in one trial and 16.3% (n = 49) had participated in two trials. Only five families reported having participated in three trials, two families in four trials, two families in five trials, and one family in seven trials. Fourteen families did not respond to the question. 46 (30.0%) of the 153 families reported participating in a clinical trial outside their country of residence.

### Children Reported Being Excluded from Participating in a Clinical Trial Abroad

Seventy-five (5.2%) of the 1,436 children were reported to be excluded from participating in a clinical trial abroad. 26 (34.7%), families reported that the exclusion was due to language barriers or the country of residence. Ten (13.3%) families reported being informed that language was one of the eligibility criteria specified in the clinical trial protocol and, for this reason, they were excluded from participation.

In 47.8% (n = 33) of the cases, the principal investigator was the person who informed the families about the exclusion. In the remaining cases, the information was provided by the patient’s physician, research nurse, or other healthcare professionals.

Regarding the reasons that motivated families to consider participating in a pediatric clinical trial abroad, 37.5% (n = 24) reported that the decision was driven by the opportunity to access a new experimental treatment not yet available in their country of residence. 32.8% (n = 21) were motivated by potential access to a new treatment that was not available through a similar clinical trial in their home country, 10.9% (n = 7) cited the opportunity to access a European-level center of excellence, and 18.7% (n = 12) reported other reasons.

### Parents’ Reports of Their Experience of Their Child Participating in a Pediatric Clinical Trial Abroad

**Fig. 1:**
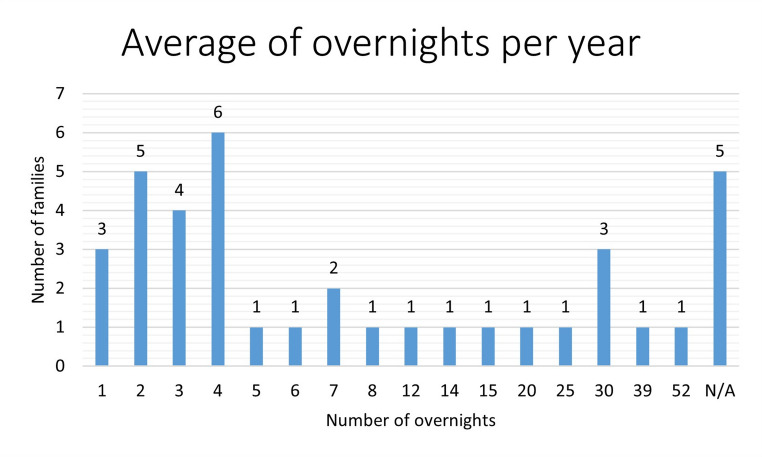
Distribution of the number of overnight stays per family per year

Thirty-eight (82.6%) of 46 families who participated in a clinical trial abroad stayed overnight during medical visits related to the trial (range 1–52 nights, mean 15,19 ± 14.67 SD). Of these, 47.4% (n = 18) stayed in a hotel, 28.9% (n = 11) stayed at the hospital, and 23.7% (n = 9) stayed in another type of accommodation. The average number of overnight stays per year varied among families. 15.8% (n = 6) families reported staying three nights per year, 13.2% (n = 5) reported staying two nights, and 10.5% (n = 4) reported staying four nights. See Fig. 1.

Considering the variability in the number of overnight stays per family during a year of participation in a clinical trial abroad, several statistical parameters were analyzed: minimum value (19), maximum value (52), standard deviation (14.67), and mean (15.19). These variables were explored to assess the diversity of study designs and to estimate the length of time families would need to stay in the foreign country where the trial was conducted. The number of days is an indicator of participant burden, particularly given that some families, particularly given that some families might not speak the country’s official language.

Forty of 46 families (86.9%) who participated in a clinical trial abroad reported on the translation of the informed consent document and about the process to access to the content provided by the sponsor of the trial. Table 5 summarizes the different translation methods used. The most common approach was translation by a trained interpreter and signing a consent form in the local language (22.5%). In six of the 46 families (13.1%), it was not necessary to translate the informed consent form, as they were able to understand and communicate in the official language of the country where the clinical trial was conducted.

**Table 5:** Methods used to translate informed consent forms for international participants

Options of the translation of the informed consent form	N =	%
Translation by a trained interpreter and signature on a consent form in the local language	9	22.5
Translation by a trained interpreter and signature on a consent form in English	7	17.5
Translation by a member of a patient organization, or family member who speaks your native language, and signature on a consent form in English	7	17.5
Translation by a digital tool such as Google translator, and signature on a consent form in the local language	2	5.0
Translation by a member of a, patient organization, or family member who speaks your native language, and signature on a consent form in the local language	1	2.5
Other	14	35.0
Total	40	100

As the survey was targeted to caregivers and young adult patients, information about the assent form and process was not included.

### Child or Parent Questionnaires or Outcome Reported Measures

Patient- and parent-reported outcome tools are commonly used in clinical trials to collect data that only participants can provide, typically related to quality of life. However, validating these tools in the native language of the patient or parents can be a barrier to including international participants in a clinical trial. Despite this limitation, some studies use professional interpreters to translate the tools, helping to make the study more inclusive and avoiding discrimination based on language. These tools are often written in simplified or “plain” language, which facilitates ad hoc oral or written translation into the patient’s language when needed.

Of the 46 families who reported having participated in a clinical study abroad, 40 provided information about the languages in which the Patient Reported Outcome Measures (PROMs) were provided. From them, seventeen families (42.5%) stated that the questionnaires were available in the patient’s native language.

Twenty families gave specific details about how they accessed the questionnaires.

In 40% of cases (n = 8), families completed the questionnaires with the assistance of a professional translator. In 35% of cases (n = 7), parents were proficient in English and completed the questionnaires in that language. Table 6 summarizes the types of translation support that enabled parents to complete the clinical trial questionnaires.

**Table 6:** Types of translations that make questionnaires accessible to international parents

Type of translation	N =	%
Professional verbal translation	8	40.0
Parents competent in English	7	35.0
Google Translator	3	15.0
Translation by other parents	1	5.0
Multilingual knowledge	1	5.0
Total	20	100

### Translation of Additional Information

Often, pediatric clinical trials provide supplementary information to patients and parents in various formats (patient diaries, flyers, websites, etc.). 39 parents replied to the question about whether any informative resources were offered as part of the clinical trial. Of these, 29 (74.4%) confirmed that additional informational materials were provided in the study in which they participated.

Among them, 51.7% accessed these resources in the original language (the official language of the site), while 48.3% had access to a translation in their respective native language.

### Parents’ Preferences About Decentralization and Digitalization of Pediatric Clinical Trials

Different decentralization and digitalization options were presented to the parents who responded to the survey, in two different theoretical settings: (1) the study was conducted in their country of residence, and (2) the clinical trial was conducted abroad.

Parents were asked to report their preferences using a Likert scale: (1) Not really willing, (2) Not willing, (3) Undecided, (4) Somewhat willing, and (5) Willing. The proposed options could potentially reduce the number of study visits and travel requirements or enhance the patient/parent experience (e.g., completing questionnaires on a digital device instead of using paper forms).

**Fig. 2:**
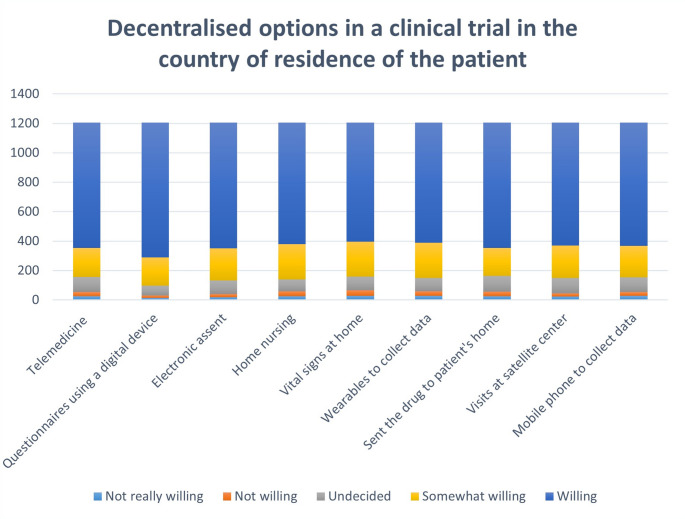
Parents' preferences regarding decentralization and digitalization when the trial is conducted in their country

**Fig. 3:**
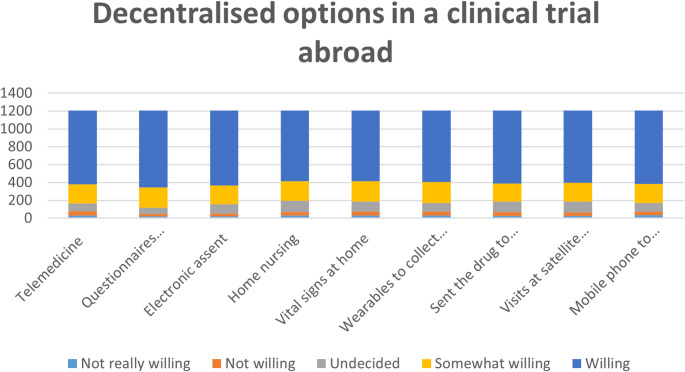
Parents' preferences regarding decentralization and digitalization when the trial is conducted abroad

In both scenarios, most (> 65%) parents indicated that they would accept any of the options. The most accepted option, both for trials conducted in the patient's country and for access to a cross-border study, was completing trial questionnaires using a digital device (76.1%). The least preferred options for studies conducted in the patient's home country were measuring vital signs at home by parents (67.2%) and using wearables to collect clinical data (67.7%). In the cross-border study scenario, the least preferred options were home nursing and having parents measure vital signs at home, with 65.5% of parents indicating they were “Willing” to accept these. See Figs. 2 and 3 Detailed data can be found in Supplementary Information.

## Discussion

We conducted a survey in Europe to explore the experiences and preferences of parents of children living with rare diseases regarding clinical trial participation, including the possibility of conducting the study abroad. The survey also explored the prospective acceptability of digitalizing and decentralizing certain clinical study activities, which may render participation less burdensome, increase flexibility, and be better adapted to the needs of families, particularly when it reduces the need for travel to the site managing the study.

To date, this represents the largest published study analyzing the experiences and preferences of parents of pediatric patients regarding cross-border access to clinical trials in Europe. A literature review identified only one previous related study published by Lalova et al. [11] in 2020, which analyzed 131 responses, 91 from representatives of patient organizations and 23 from individual patients or parents but not focused in pediatric clinical trials.

### Parent Experiences in a Cross-Border Pediatric Clinical Trial

From the sample (N = 1,436), 10.7% of families reported having participated in a clinical study (n = 153), of which 46 families (30.1%) stated that the trial was conducted outside their country of residence allowing us to explore how language accommodation was managed to enable their participation. In 22.5% of cases, the translation of the informed consent form was performed by a trained interpreter, and participants signed the version in the local language of the site where the study was conducted. In 35% of cases, participants signed the English version of the informed consent form after receiving a translation provided either by a trained interpreter, a patient organization, or a family member fluent in both English and the family's mother tongue. Regarding patient-reported outcome measures (PROMs) and quality-of-life questionnaires, 42.5% of these tools were validated and available in the patient's native language.

Currently, clinical trial participation outside the country of residence is uncommon in Europe. Traveling abroad for clinical trials places a significant burden (e.g., travel, expenses) on families, and the processes to enable such participation are poorly developed. As these processes develop, as decentralized trial elements become more common, and as the number of sites per trial decreases [12, 13], the only potential therapeutic option may be cross-border access to trials. The fact that families would travel to access a clinical trial (potential future new treatment) (37.5%) and/or for a clinical trial unavailable in their home country (32.8%) demonstrates their commitment to participate. These data demonstrate that parents of children suffering from rare diseases, of which only 5% have a targeted treatment currently, are willing to travel where any therapeutic opportunity can be offered to their child [14, 15].

### Scientific Benefits of Cross-Border Access in Pediatric Clinical Trials

Allowing the inclusion of international patients in pediatric clinical trials will increase the generalizability of study results. The patients recruited to the trial will more accurately reflect the population with the condition and helps to expose any subgroup differences in the efficacy and safety of the intervention, across biological differences (e.g., age, sex) as well as social determinants of health that may impact patient responses [16–18]. While some social determinants of health, such as nutrition status, may have biological consequences, others may result in discrimination. One of these is language, interpreted broadly as the person’s preferred language, competence in a different language, language proficiency, or communication access [19, 20].

### Language as a Social Determinant of Health: Upholding European Patient Rights Against Discrimination

In Europe, specific regulations protect patients from discrimination based on language, notably article 2 of the *Universal Declaration of Human Rights* (UDHR, 1948) [21], article 21 and 22 of the *Charter of Fundamental Rights of the European Union* (2000)[22] and article 21 in the *United Nations Convention on the Rights of the Child* (1989) [23]. In alignment with these regulations, and to uphold the ethical principles of clinical research [24] including the principles of equity and inclusion [25],_we encourage research teams and the broader scientific community to incorporate a diversity plan when designing clinical trial protocols. This plan should be aimed at the analysis of the epidemiology of the population that has the condition, biological variables, and social determinants of health, including language.

### English Language Proficiency Among Parents of Children Living with Rare Diseases

While language accommodation can be provided and tailored to specific needs, a challenging barrier is the lack of validated Patient-Reported Outcome Measures (PROMs) and Quality of Life (QoL) scales in the many languages of the patients or their parents. Specifically, regarding the language competence of parents of children living with a rare disease, our survey showed that 800 parents (55.7%) reported being able to communicate in English, but of variable competencies (see Supplementary Information).

Nevertheless, the competence in English as a second language, most parents of children with a rare disease (75.5%) are proficient in English and capable of understanding and communicating in this language, can help overcome the absence of validated tools in the patient’s or parent’s native language. In cases where this is not feasible, personal experience has shown that some sponsors are willing to provide professional translations of these tools into the patient’s or parent’s native language.

### Language Discrimination in Accessing Cross-Border Pediatric Clinical Trials

Despite these accommodations, language-based discrimination still occurs, preventing participation in clinical trials. In the European sample analyzed here, 26 families (34.7%) reported being excluded from participating abroad in a clinical trial due to language barriers or country of residence. Additionally, 10 families (13.3%) indicated awareness that language was listed as an eligibility criterion in the clinical trial protocol. Further research is needed to determine whether the language-based eligibility exclusion criteria are scientifically justified.

The sample (N = 1,436) accessed here is limited. In response, and in order to collect data prospectively, we have developed a tool for clinical trials units (CTUs) to report cases of language-based ineligibility criteria or cross-border access challenges. Beyond pediatric clinical trials, similar challenges exist in cross-border healthcare access for patients with rare and ultra-rare diseases, particularly in low- and middle-income countries in the European regulatory landscape (e.g., Ukraine), in European member states (e.g., Bosnia Herzegovina) and in other regulatory regions [26, 27]. If international patients are not considered for trial participation across different regulatory regions, this may negatively impact recruitment—particularly in pediatric ultra-rare diseases, where enrollment is intrinsically challenging—and extend the time required to complete the trial.

### Digitalization and Decentralization of Pediatric Clinical Trials

When accessing cross-border pediatric clinical trials, options such as digital health technologies can reduce the burden, while telemedicine, local clinical sites, or home nursing can decrease the need to travel to the main clinical trial site. Parents are willing to embrace opportunities to digitalize and decentralize the conduct of pediatric clinical trials, whether conducted in the country of residence or abroad. Of the various elements of decentralization and digitalization presented in our survey, completing study questionnaires using digital tools was the most accepted. And while this option does not travel, it supports the deployment of digital technologies in clinical trials. Conducting visits at a satellite center was well accepted (66.9%) as was sending the investigational drug to the patient’s home (67.8%), particularly when studies were conducted abroad.

We therefore recommend that pediatric clinical trials should be designed to include digital technologies, remote visits, and other elements of decentralization that will reduce burden on families, informed by an assessment of families’ needs, expectations, and preferences. In this context, a plan to accommodate language preferences in the use of wearables, digital platforms, and other innovations should be established during protocol design. Each clinical trial protocol will require a specific decentralization and digitalization plan based on its specific aspects (e.g., disease, patient age, type of design, etc.).

### Limitations

The results and generalizability of the research is limited by the number of survey responses completed. To our knowledge, this is the first published data to query cross-border access to clinical trials analyzing the experience and preferences of parents of children living with rare diseases across Europe. Despite this, the data cannot be considered to be representative across the different European countries. The fact that 44.2% of responses came from Spanish and Catalan speakers is indicative of selection bias. Comparisons between and across countries are not possible.

We cannot determine whether the data collected from parents with prior experience in cross-border clinical trials are statistically robust, given the limited number of respondents and the fact that the denominator of the number of patients who have traveled to another EU Member State to participate in a clinical trial is unknown. However, the analysis here of the 46 cases collected from families who accessed a cross-border clinical trial compares favorably to the number of patient requests to access cross-border healthcare services. According to the European Commission’s report “*Member State data on cross-border patient healthcare following Directive 2011/24/EU – Reference year 2023*” (published February 2025), only 5,973 requests were received from 15 European countries, of which 4,838 were approved. These requests were concentrated in just three countries: Germany (3910), Luxembourg (992), and Slovakia (619), accounting for 92.4% of the total. Compared with this data, the analysis of the 46 cases collected from families who accessed a cross-border clinical trial can be considered relevant.

Further research is needed to obtain a larger sample of responses from families across different European countries. This would allow for country-level comparisons and provide further insight into operational needs, including how language accommodation was managed. Our study demonstrates that language accommodation is feasible, barriers can be overcome, and efforts to increase access to pediatric clinical trials should be prioritized, particularly in response to unmet medical needs.

## Conclusion

This study provides the first European analysis of the experience and preferences of parents of children living with rare diseases and cross-border access to clinical trials. Despite limitations in sample size and geographic representation, the findings offer valuable insights into the motivation, barriers, and facilitators that influence family decisions to participate in clinical trials abroad. Our data confirms that families are willing to travel internationally when clinical trials represent the only available therapeutic option, particularly in the context of ultra-rare diseases, highlighting the ethical imperative to ensure equitable access to research, regardless of language, country of residence, or socioeconomic status.

Our findings demonstrate that language accommodation is feasible, even in the absence of validated tools in the patient’s preferred language. Many parents have sufficient English proficiency to participate meaningfully, and when they do not, professional translation services have been and can be provided.

Language should only be used as an eligibility criterion when scientifically justified, such as in studies of the neurodevelopmental acquisition of language. Cross-border access to pediatric clinical trials, and language accommodation, will help ensure that the rights of children and patients are respected.

## Supplementary Information

Below is the link to the electronic supplementary material.

Supplementary file1 (DOC 292 KB)

## Data Availability

Full data is available on request.

## Footnotes

1 The dominance of Spanish and Catalan likely reflect the influence of the principal author (see Discussion).
