# Supplementary material for: Parents' Perspectives on Access to Pediatric Rare Disease Cross-Border Clinical Trials in Europe: Experiences of Language Inclusion and Preferences
Source: Ther Innov Regul Sci. 2026 Mar 18;60(4):1037–48. doi: 10.1007/s43441-026-00942-y (PMC13354654; doi:10.1007/s43441-026-00942-y)
Supplement: Supplementary file 1 — Supplementary file1 (DOC 292 KB) [file 43441_2026_942_MOESM1_ESM.doc]

**SUPPLEMENTARY INFORMATION**

Type of residence of the survey respondents

|  | **N=** | **%** |
| --- | --- | --- |
| Living in the country where the patient was born | 1,272 | 88.58% |
| Permanent residency / double nationality | 52 | 3.62% |
| Permanent residency / Emigrant | 47 | 3.27% |
| Temporary residency (studies/work…) | 13 | 0.91% |
| Asylum | 1 | 0.07% |
| Other | 51 | 3.55% |
| **Totals** | **1,436** | **100.00%** |

|  | | | |  | |  | | | |  | |  | | |
| --- | --- | --- | --- | --- | --- | --- | --- | --- | --- | --- | --- | --- | --- | --- |
| Country where the respondent was born | | | |  | | Country where the patient was born | | | |  | | Country of residence | | |
| **Country** | **N=** | **%** |  | | **Country** | | **N=** | **%** |  | | **Country** | | **N=** | **%** |
| Spain | 594 | 41.36% |  | | Spain | | 609 | 42.41% |  | | Spain | | 619 | 43.11% |
| France | 114 | 7.94% |  | | France | | 109 | 7.59% |  | | France | | 112 | 7.80% |
| Other | 112 | 7.80% |  | | Other | | 81 | 5.64% |  | | Slovenia | | 92 | 6.41% |
| Slovenia | 85 | 5.92% |  | | Slovenia | | 88 | 6.13% |  | | Portugal | | 79 | 5.50% |
| Poland | 84 | 5.85% |  | | Poland | | 71 | 4.94% |  | | Poland | | 78 | 5.43% |
| Portugal | 69 | 4.81% |  | | Portugal | | 72 | 5.01% |  | | Germany | | 72 | 5.01% |
| Italy | 63 | 4.39% |  | | Italy | | 60 | 4.18% |  | | Other | | 61 | 4.25% |
| Germany | 61 | 4.25% |  | | Germany | | 70 | 4.87% |  | | Italy | | 59 | 4.11% |
| Croatia | 40 | 2.79% |  | | Croatia | | 41 | 2.86% |  | | Croatia | | 41 | 2.86% |
| United Kingdom | 36 | 2.51% |  | | United Kingdom | | 42 | 2.92% |  | | United Kingdom | | 38 | 2.65% |
| Greece | 33 | 2.30% |  | | Greece | | 36 | 2.51% |  | | Greece | | 34 | 2.37% |
| Czech Republic | 21 | 1.46% |  | | Czech Republic | | 23 | 1.60% |  | | Czech Republic | | 24 | 1.67% |
| Ireland | 16 | 1.11% |  | | Ireland | | 22 | 1.53% |  | | Ireland | | 20 | 1.39% |
| Romania | 14 | 0.97% |  | | Romania | | 7 | 0.49% |  | | Belgium | | 16 | 1.11% |
| The Netherlands | 13 | 0.91% |  | | The Netherlands | | 11 | 0.77% |  | | Switzerland | | 15 | 1.04% |
| Belgium | 11 | 0.77% |  | | Belgium | | 15 | 1.04% |  | | Finland | | 11 | 0.77% |
| Finland | 10 | 0.70% |  | | Finland | | 10 | 0.70% |  | | The Netherlands | | 11 | 0.77% |
| Hungary | 10 | 0.70% |  | | Hungary | | 10 | 0.70% |  | | Hungary | | 9 | 0.63% |
| Bosnia and Herzegovina | 7 | 0.49% |  | | Bosnia and Herzegovina | | 3 | 0.21% |  | | Romania | | 7 | 0.49% |
| Bulgaria | 7 | 0.49% |  | | Bulgaria | | 5 | 0.35% |  | | Andorra | | 6 | 0.42% |
| Denmark | 5 | 0.35% |  | | Denmark | | 2 | 0.14% |  | | Bulgaria | | 5 | 0.35% |
| Norway | 5 | 0.35% |  | | Norway | | 5 | 0.35% |  | | Norway | | 5 | 0.35% |
| Sweden | 5 | 0.35% |  | | Sweden | | 7 | 0.49% |  | | Sweden | | 5 | 0.35% |
| Switzerland | 5 | 0.35% |  | | Switzerland | | 16 | 1.11% |  | | Austria | | 3 | 0.21% |
| Slovakia | 3 | 0.21% |  | | Slovakia | | - | 0.00% |  | | Denmark | | 3 | 0.21% |
| Cyprus | 3 | 0.21% |  | | Cyprus | | 1 | 0.07% |  | | Latonia | | 2 | 0.14% |
| Andorra | 2 | 0.14% |  | | Andorra | | 5 | 0.35% |  | | Bosnia and Herzegovina | | 1 | 0.07% |
| Iceland | 2 | 0.14% |  | | Iceland | | 2 | 0.14% |  | | Cyprus | | 1 | 0.07% |
| Austria | 1 | 0.07% |  | | Austria | | 3 | 0.21% |  | | Estonia | | 1 | 0.07% |
| Estonia | 1 | 0.07% |  | | Estonia | | 1 | 0.07% |  | | Iceland | | 1 | 0.07% |
| Latonia | 1 | 0.07% |  | | Latonia | | 1 | 0.07% |  | | Malta | | 1 | 0.07% |
| North Macedonia | 1 | 0.07% |  | | North Macedonia | | 1 | 0.07% |  | | North Macedonia | | 1 | 0.07% |
| Malta | 1 | 0.07% |  | | Malta | | 1 | 0.07% |  | | Slovakia | | 1 | 0.07% |
| Ukraine | 1 | 0.07% |  | | Ukraine | | 2 | 0.14% |  | | Ukraine | | 1 | 0.07% |
|  | **1,436** | **100.00%** |  | | Kosovo | | 1 | 0.07% |  | | Liechtenstein | | 1 | 0.07% |
|  |  |  |  | | Luxemburg | | 3 | 0.21% |  | | Kosovo | |  | 0.00% |
|  |  |  |  | | **Total** | | **1,436** | **100.00%** |  | | Luxemburg | | 0 | 0.00% |
|  |  |  |  | |  | |  |  |  | | **Total** | | **1,436** | **100.00%** |

Native languages of respondents

| **Languages** | **Number of answers** | **%** |
| --- | --- | --- |
| Spanish | 545 | 37.95% |
| French | 124 | 8.64% |
| Catalan | 90 | 6.27% |
| Portuguese | 87 | 6.06% |
| Slovenian | 85 | 5.92% |
| Polish | 82 | 5.71% |
| German | 66 | 4.60% |
| Italian | 65 | 4.53% |
| English | 57 | 3.97% |
| Croatian | 46 | 3.20% |
| Other | 46 | 3.20% |
| Greek | 36 | 2.51% |
| Czech | 21 | 1.46% |
| Dutch | 18 | 1.25% |
| i Romanian | 13 | 0.91% |
| Hungarian | 12 | 0.84% |
| Finish | 10 | 0.70% |
| Bulgarian | 6 | 0.42% |
| Norwegian | 5 | 0.35% |
| Swedish | 5 | 0.35% |
| Danish | 4 | 0.28% |
| Slovak | 3 | 0.21% |
| Irish | 3 | 0.21% |
| Icelandic | 3 | 0.21% |
| Estonian | 2 | 0.14% |
| Latvian | 1 | 0.07% |
| Maltese | 1 | 0.07% |
| Serbian | - | 0.00% |
|  | **1,436** | **100.00%** |

| Parents able to communicate in English | | |  | Level of English of the parents: | | |
| --- | --- | --- | --- | --- | --- | --- |
|  | **N=** | **%** |  |  | **N=** | **%** |
| Yes | 800 | 55,71% |  | Beginner | 85 | 10,63% |
| No | 636 | 44,29% |  | Intermediate | 310 | 38,75% |
|  | **1436** | **100,00%** |  | Advance | 294 | 36,75% |
|  |  |  |  | Native | 111 | 13,88% |
|  |  |  |  |  | **800** | 100,00% |
|  |  |  |  |  |  |  |
| Patient fluent in English | | |  | Level of English of the patient | | |
|  | **N=** | **%** |  |  | **N=** | **%** |
| Yes | 211 | 14,69% |  | Beginner | 33 | 15,64% |
| No | 1221 | 85,03% |  | Intermediate | 62 | 29,38% |
| N/A | 4 | 0,28% |  | Advance | 51 | 24,17% |
|  | **1436** | **99,72%** |  | Native | 65 | 30,81% |
|  |  |  |  |  | **211** | 100,00% |
|  |  |  |  |  |  |  |
| Can you understand and make yourself understood in a language other than English and your native language? | | |  |  |  |  |
|  | **N=** | **%** |  |  |  |  |
| Yes | 547 | 38,09% |  |  |  |  |
| No | 889 | 61,91% |  |  |  |  |
|  | **1436** | **100,00%** |  |  |  |  |

**Parents' preferences regarding decentralization and digitalization when the trial is conducted in their country**

|  | **Not really willing** | **Not**  **willing** | **Undecided** | **Somewhat willing** | **Willing** |
| --- | --- | --- | --- | --- | --- |
| Telemedicine | 1.99% | 2.49% | 8.39% | 16.36% | 70.76% |
| Questionnaires using a digital device | 1.08% | 1.50% | 5.40% | 15.95% | 76.08% |
| Electronic assent | 1.41% | 1.83% | 7.72% | 18.11% | 70.93% |
| Home nursing | 2.08% | 2.82% | 6.73% | 19.93% | 68.44% |
| Vital signs at home | 2.24% | 3.24% | 7.64% | 19.68% | 67.19% |
| Wearables to collect data | 2.24% | 2.57% | 7.48% | 19.93% | 67.77% |
| Sent the drug to patient's home | 2.08% | 2.66% | 8.80% | 15.78% | 70.68% |
| Visits at satellite center | 1.91% | 1.66% | 8.80% | 18.27% | 69.35% |
| Mobile phone to collect data | 2.24% | 2.24% | 8.22% | 17.86% | 69.44% |

Parents' preferences regarding decentralization and digitalization when the trial is conducted abroad

|  | **Not really willing** | **Not willing** | **Undecided** | **Somewhat willing** | **Willing** |
| --- | --- | --- | --- | --- | --- |
| Telemedicine | 2.57% | 3.74% | 7.39% | 17.69% | 68.60% |
| Questionnaires digital device | 1.74% | 2.24% | 5.90% | 18.60% | 71.51% |
| Electronic assent | 1.91% | 2.33% | 8.72% | 17.44% | 69.60% |
| Home nursing | 2.33% | 3.41% | 10.38% | 18.36% | 65.53% |
| Vital signs at home | 2.33% | 3.82% | 9.39% | 18.94% | 65.53% |
| Wearables to collect data | 2.57% | 3.41% | 8.22% | 19.52% | 66.28% |
| Sent the drug to patient's home | 1.99% | 3.65% | 9.88% | 16.69% | 67.77% |
| Visits at satellite center | 2.16% | 3.32% | 10.13% | 17.52% | 66.86% |
| Mobile phone to collect data | 2.91% | 3.16% | 7.97% | 17.77% | 68.19% |

**Appendix 1**


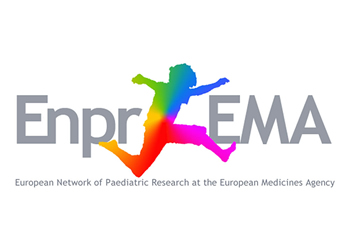


**QUESTIONNAIRE ADDRESSED TO PARENTS OF CHILDREN LIVING WITH A DISEASE**

Thank you for your interest in participating in this research initiative promoted by the European Network of Paediatric Research at the European Medicines Agency (Enpr-EMA). Your contribution is important to provide us with necessary clinical information to analyse:

1. how the access to paediatric patients to cross-border clinical trials in Europe is managed in terms of language barriers, and
2. If you have no experience of participating in a clinical trial, we would like to know your preferences.

Please take into account that this research project is addressed to all parents who are willing to contribute and who meet these criteria:

- **Age of patients**: from 0 to 17 years old
- **All diseases, disorders, conditions or syndromes** diagnosed in children and young people are eligible in the data collection.
- **Clinical trials focused on medicines** (in phases I, II, III, and IV).
- Not only patients with experience travelling from abroad to **participate in a clinical trial in a European country** can answer this questionnaire.
- You can also complete the questionnaire **if the patient has participated in a clinical trial in the country where you live but this country is not your country of origin**.
- The term **native language,** also referred to as **mother tongue** in this questionnaire refers to the language() that a person acquires in early childhood because they are spoken at home

1. **IMPORTANT: Remember that in the case that you do not have experience participating in a clinical trial, we would like to know about your preferences.**

**Completion of this survey takes about 12-15 minutes. Your contribution will be relevant to help in the process to create future guidance and recommendations to facilitate the inclusion of children and young people in cross-border clinical trials across Europe, with no exclusion about their mother tongue.**

The team involved in this research truly appreciate your time and help.

Enpr-EMA Cross-border access to clinical trials Working Group (WG)

Begonya Nafria, Chair of the WG

[begonya.nafria@sjd.es](mailto:begonya.nafria@sjd.es)

This work is done by the Working Group of Paediatric Clinical Trials Cross-border Access in Europe, operating under the European Network of Paediatric Research at the European Medicines Agency (Enpr-EMA).

This Working Group leader is Begonya Nafria (PhD Candidate ) from Sant Joan de Déu Children´s Hospital in Spain, and all the data and information produced for this particular Working Group have been approved by the Sant Joan de Déu Research Foundation's Ethics Committee.

**Data protection:**

*The answers to this questionnaire are anonymous. The data is saved at the Sant Joan de Déu Children´s Hospital for data analysis purposes and for the required time to do aggregated analysis of this survey*. *In case you want to change, remove or cancel the already completed survey, please contact the leadership team. begonya.nafria@sjd.es*

**TELL US A BIT ABOUT YOU**

*This information will be important when we analyse all the responses that we collect.*

Who are you? (*)

- Father
- Mother
- Other caregiver (please specify: ___________)

You are: XXX years old (*)

Current age of the patient: XXX years old (*)

Country where you were born (*):

Albania, Andorra, Austria, Belgium, Bosnia and Herzegovina, Bulgaria, Croatia, Republic of Cyprus, Czech Republic, Denmark, Estonia, Finland, France, Germany, Greece, Hungary, Ireland, Iceland, Italy, Kosovo, Latvia, Liechtenstein, Lithuania, Luxembourg, Malta, Montenegro, Netherlands, North Macedonia, Norway, Poland, Portugal, Romania, Slovakia, Slovenia, Spain, Sweden, Switzerland, Ukraine, United Kingdom… other

Country where the patient was born (*):

Albania, Andorra, Austria, Belgium, Bosnia and Herzegovina, Bulgaria, Croatia, Republic of Cyprus, Czech Republic, Denmark, Estonia, Finland, France, Germany, Greece, Hungary, Ireland, Iceland, Italy, Kosovo, Latvia, Liechtenstein, Lithuania, Luxembourg, Malta, Montenegro, Netherlands, North Macedonia, Norway, Poland, Portugal, Romania, Slovakia, Slovenia, Spain, Sweden, Switzerland, Ukraine, United Kingdom… other

Country where you are currently living (*):

Albania, Andorra, Austria, Belgium, Bosnia and Herzegovina, Bulgaria, Croatia, Republic of Cyprus, Czech Republic, Denmark, Estonia, Finland, France, Germany, Greece, Hungary, Ireland, Iceland, Italy, Kosovo, Latvia, Liechtenstein, Lithuania, Luxembourg, Malta, Montenegro, Netherlands, North Macedonia, Norway, Poland, Portugal, Romania, Slovakia, Slovenia, Spain, Sweden, Switzerland, Ukraine, United Kingdom… other

If you are living in a country different from your country of origin, please select the option that applies:

- Non applicable / We live in the country were the patient was born
- Temporary residence (due to work or studies)
- Permanent residence - immigrant
- Permanent residence - double nationality –
- Refugee
- Asylum
- Other (please specify: ___________)

Disease that the patient is living with, or disease that the patient has had (e.g. cancer survivor) (*):

Is this a rare disease? (*) □ Yes □ No □ I don’t know

Select the medical departments that currently are taking care of the patient (*):

- Adolescent Medicine
- Adolescent Psychiatry
- Allergy and Immunology
- Anaesthesiology
- Bone marrow Transplantation
- Behavioural Medicine
- Cardiology
- Cardiovascular Surgery
- Child Psychiatry
- Clinical Genetics
- Critical Care / Emergency Medicine
- Dentistry and Orthodontics
- Dermatology
- Developmental and Behavioural medicine
- Diagnostic Imaging
- Emergency
- Epidemiology
- Endocrinology /Diabetes
- Gastroenterology
- General Paediatrics
- Gynaecology, Obstetrics
- Haematology
- Hepatology
- Infectious Diseases
- Intensive Care
- Mental Health
- Metabolic Diseases
- Neonatology
- Nephrology
- Neurology and Neurophysiology
- Neurosurgery
- Nutrition
- Oncology
- Ophthalmology
- Paediatric Orthopaedics
- Otolaryngology
- Pain Management
- Palliative Care
- Paediatric Rehabilitation
- Public Health
- Primary Paediatrics
- Physical Rehabilitation
- Pulmonology and Asthma
- Rheumatology
- Sports medicine
- Sexual abuse and Child abuse
- Solid organ Transplantation
- Paediatric Surgery
- Urology
- Traumatology
- Vaccinology

Which is your mother tongue (*):

Bulgarian, Catalan, Croatian, Czech, Danish, Dutch, English, Estonian, Finnish, French, German, Greek, Hungarian, Icelandic, Irish, Italian, Latvian, Lithuanian, Luxembourgish, Maltese, Norwegian, Polish, Portuguese, Romanian, Slovak, Slovenian, Spanish, Swedish, other

Are you able to communicate in English? Yes/No

How would you describe your level of English? Beginner / Intermediate / Advanced / Native-like

Is the patient able to communicate in English? Yes/No

How would you describe the patient’s level of English? Beginner / Intermediate / Advanced / Native-like

Can you understand and make yourself understood in a language other than English and your mother tongue? (*)? Yes/No

If so, in which language(s) different than your mother language:

Bulgarian, Catalan, Croatian, Czech, Danish, Dutch, English, Estonian, Finnish, French, German, Greek, Hungarian, Icelandic, Irish, Italian, Latvian, Lithuanian, Luxembourgish, Maltese, Norwegian, Polish, Portuguese, Romanian, Slovak, Slovenian, Spanish, Swedish, other

Can the patient understand and make him/herself understood in a language other than English (*)? Yes/No

If so, in which language(s) different than your mother language:

Bulgarian, Catalan, Croatian, Czech, Danish, Dutch, English, Estonian, Finnish, French, German, Greek, Hungarian, Icelandic, Irish, Italian, Latvian, Lithuanian, Luxembourgish, Maltese, Norwegian, Polish, Portuguese, Romanian, Slovak, Slovenian, Spanish, Swedish, other

**EXPERIENCE AS PARTICIPANT IN A PAEDIATRIC CLINICAL TRIAL**

If the patient has ever participated in a clinical trial, we need some information to know how it was performed.

Has the patient ever participated in a clinical trial (CT) (*)? Yes/No

If so, how many clinical trials has the patient been a part of?

If so, complete the following information about the last or the most complex clinical trial in which the patient participated:

- What is the CT identifier/ EUDRA-CT or CTIS code? (You can find this information in: [www.clinicaltrials.gov](http://www.clinicaltrials.gov/))
- Is the patient currently participating in the clinical trial? Yes/No
  - If so, how long has it been since s/he started? XXX months
- Total duration of the trial in months:
- Average number of visits to hospital for the trial per year (approx.):
- Route of administration of the treatment:
  - Oral
  - Ophthalmic (eye)
  - Otic (ear)
  - Nasal
  - Inhaled (pulmonary)
  - Topical (skin)
  - Parenteral (injection or infusion)
  - Rectal
  - Vaginal
- The experimental treatment of the clinical trial, was administered in the site? Yes/No

Disease/condition/symptom of the clinical trial. Please, provide any information that you may have:[Name] (*)

If you know, identify the study type (select all that apply, i.e., Phases I/II):

- Early phase I
- Phase I
- Phase II
- Phase III
- I don’t know/ not sure

Type of study:

- Academic
- Commercial (led by a pharma company)
- Not sure/Don’t know

Has the patient travelled abroad to participate in any part of this clinical trial? Yes/No

If so, to which country?

**EXPERIENCE IF THE PATIENT WAS NOT ABLE TO TAKE PART IN A PAEDIATRIC CLINICAL TRIAL ABROAD**

If the patient has ever been excluded from participating in a clinical trial abroad, we need some information in order to assess if the exclusion was based on scientific criteria.

Has the patient ever been excluded from participating in a clinical trial abroad? (*) Yes/No

- If so, do you know why? [Open answer question]
- Do you know if the patient’s language was an exclusion criterion in the clinical trial protocol?
  - Yes, excluded because of mother tongue
  - No , he/she was not excluded through mother tongue
  - Don't know
- Who informed you that the patient would not be allowed to take part in the clinical trial?
  - - Principal investigator or person in charge of the clinical trial
    - Research nurse
    - The patient’s doctor
    - Another professional (please specify: ____ _____ )
- Can you provide information about the clinical trial the patient was excluded from?
- CT identifier/ EUDRA-CT or CTIS code. You can find this information in: [www.clinicaltrials.gov](http://www.clinicaltrials.gov/))
- Disease/condition/symptom of the clinical trial [Name]

Which factor(s) prompted your decision to participate in a clinical trial abroad?

- Access to a new treatment that is not marketed in my country of residence
- Access to a new treatment that is not available in a similar clinical trial in my country of residence
- Access to a new treatment that is too expensive in my country of residence
- Access to a centre of Excellence at the European level
- Other (please specify: ________________---)

**EXPERIENCE AS PARTICIPANT IN A CLINICAL TRIAL ABROAD**

If the patient has ever participated in a clinical trial abroad, we need some information to know about their experience.

Has the patient travelled abroad to participate in a clinical trial? Yes/No

Has the patient stayed overnight during the medical visits of the trial? Yes/No

- If so, where did the patient stay?
  - Hospital
  - Hotel
  - Other (detail)
- If so, how many nights per visit did s/he stay overnight on average per year?

Did the patient drop out of the study due to travel distance/time, travel costs, family inconvenience, or absence from work/being dismissed from work? Yes/No

If so, please explain (open field)

Which factor(s) prompted your decision to participate in a clinical trial abroad?

- Access to a new treatment that is not marketed in my country of residence
- Access to a new treatment that is not available in a similar clinical trial in my country of residence
- Access to a new treatment that is too expensive in my country of residence
- Access to a centre of Excellence at the European level
- Other (please specify: ­­­­­­­­­­­­______________)

**Consent and Assent**

We would like to know about the study and how you signed the consent form to take part.

Select the right option in regards to the documents on consent you had access to:

- Translation by a trained interpreter and signature on a consent form in English
- Translation by a trained interpreter and signature on a consent form in the local language
- Translation by a member of a patient organisation, or family member who speaks your native language, and signature on a consent form in English
- Translation by a member of a, patient organisation, or family member who speaks your native language, and signature on a consent form in the local language
- Translation by a digital tool such as Google translator, and signature on a consent form in the local language

**Questionnaires asking you about your health**

During the trial you or the patient had to complete questionnaires regarding the symptoms, health status, and other relevant information.. Below you will find questions about your access to these tools.

Were the questionnaires available in the mother tongue of the patient? Yes/No

- If not, can you explain the process that you followed in order to complete the information that the clinical study required? (open field)

Do you remember the name of the questionnaires you were asked to complete (name of the scale or questionnaire)?

Other information addressed to the patients involved in the clinical trial

Did the trial offer informative resources for the patients (booklets, patient diaries, etc.)? Yes/No

Which type of resources were available?

- Information booklets
- Patient diaries
- Electronic patient diaries
- Multimedia resources (videos, games, etc.)
- App
- Website
- Other (s) Please, detail:

How did you access these resources?

- Translation into my mother tongue
- In their original language

If you would like to provide additional information about your experience dealing with documentation and tools of the clinical trial which were not accessible in your mother tongue, please share below:

**Patient and family preferences as regards cross-border access and decentralised clinical trials.**

*Some activities of clinical trials can be performed with the implementation of technology. In this section, we will ask about your preferences.*

Would you be willing to engage in the following during a clinical trial performed in the country of the patient?

(Likert scale: Not willing / Not really willing / Undecided / Somewhat willing / Willing)

- Telemedicine visits (i.e., remote visits using teleconference technology)
- Access to questionnaires and quality of life scales via a digital device (e.g., mobile phone or tablet)
- Electronic assent and/or electronic consent
- Home nursing to perform some medical procedures (e.g., blood tests, vital signs, etc.)
- Patient or caregiver to take vital signs at home (e.g. blood pressure, glucose level, etc.).
- Use of wearables to collect medical data (e.g., smart watch to monitor blood pressure or heart rate)
- Having the medicine needed during the clinical trial sent home (i.e., no need to travel to the site)
- Having some medical procedures or visits at a satellite site closer to the patient’s home
- Use of your personal mobile phone to collect data during participation in the clinical trial

Would you be willing to engage in the following during a clinical trial performed abroad?

(Likert scale: Not willing / Not really willing / Undecided / Somewhat willing / Willing)

- Telemedicine visits (i.e., remote visits using teleconference technology)
- Access to questionnaires and quality of life scales via a digital device (e.g., mobile phone or tablet)
- Electronic assent and/or electronic consent
- Home nursing to perform some medical procedures (e.g., blood tests, vital signs, etc.)
- Patient or caregiver to take vital signs at home (e.g. blood pressure, glucose level, etc.).
- Use of wearables to collect medical data (e.g., smart watch to monitor blood pressure or heart rate)
- Having the medicine needed during the clinical trial sent home (i.e., no need to travel to the site)
- Having some medical procedures or visits at a satellite site closer to the patient’s home

Use of your personal mobile phone to collect data during participation in the clinical trial

If you want to tell us more with a brief interview on this topic, please contat us by email: begonya.nafria@sjd.es

**Thank you for taking the time to complete this questionnaire. Your contribution will help us learn about language and cultural discrimination in accessing clinical trials abroad, good practices, and patients’ preferences.**

**If your son or daughter is 12 years old or older, we would appreciate it if you could give your consent for their participation in a dedicated survey addressed to young patients by accessing this** [**link**](https://sjdhospitalbarcelona.qualtrics.com/jfe/form/SV_eUOBbOaRY1QSubQ)**.**

Thank you so much for participating in this initiative!

Access, rectification or deletion of data, as well as other rights, detailed in the Spanish Organic Law for the Protection of Personal Data (LOPD) and in accordance with Regulation (EU) 2019/679 of the European Parliament and the Council of 27 April 2016 on the protection of people with respect to the processing of personal data as well as the transfer of said data (GDPR), has to be executed through a written communication to begonya.nafria@sjd.es
